# Temporal dynamics of polar bear (*Ursus maritimus*) pregnancy rates in western Hudson Bay: influence of mass, age and timing of first breeding

**DOI:** 10.1093/conphys/coaf058

**Published:** 2025-08-02

**Authors:** David McGeachy, Nicholas J Lunn, Evan S Richardson, Andrew E Derocher

**Affiliations:** Wildlife Research Division, Science and Technology Branch, Environment and Climate Change Canada, 11455 Saskatchewan Dr. Edmonton, AB T6G 2E9, Canada; Department of Biological Sciences, University of Alberta, 11455 Saskatchewan Dr. Edmonton, AB T6G 2E9, Canada; Wildlife Research Division, Science and Technology Branch, Environment and Climate Change Canada, 11455 Saskatchewan Dr. Edmonton, AB T6G 2E9, Canada; Wildlife Research Division, Science and Technology Branch, Environment and Climate Change Canada, 123 Main St. Winnipeg, MB R3C 4W2, Canada; Department of Biological Sciences, University of Alberta, 11455 Saskatchewan Dr. Edmonton, AB T6G 2E9, Canada

**Keywords:** First breeding, hormone, polar bears, pregnancy rates, progesterone, reproduction, sea ice

## Abstract

Reproduction is the most energetically costly undertaking for female mammals and for capital breeders. Understanding factors that influence individual body condition and reproductive success is essential to understanding population demography. We investigated long-term trends in pregnancy rates to assess the impacts of individual and environmental factors on polar bear reproduction. Pregnancy status was determined from serum progesterone levels in blood collected from free-ranging polar bears captured on shore in late summer to early autumn in western Hudson Bay, Canada. We analysed 541 blood samples for progesterone level from 441 individuals from 1991 to 2021 and compared to data from 1982 to 1990 (354 individuals from 476 occasions). We used a generalized linear model to investigate individual and environmental factors that could influence pregnancy rates. The percent of solitary females that were pregnant declined significantly over time and between time periods from 85% in 1982–90 to 73% in 1991–2021. Interannual variation in pregnancy was high, ranging from 46 to 100%. Pregnancy rates were influenced by mass and age, with higher pregnancy rates for heavier females and those >4 and <24 years old. The percentage of pregnant 4-year-old females declined from 82% in 1982–90 to 55% in 1991–2021. The mass of pregnant females declined over time and the lightest pregnant female known to have produced cubs weighed 196 kg in the autumn. We suggest further research is needed to understand mechanisms resulting in pregnancy rate variation, which may be related to previous reproductive status and recent litter loss.

## Introduction

Reproductive dynamics have significant effects on population demography and are influenced by life history evolution and environmental conditions (Roff, 1992). Successful reproduction in female mammals is energetically demanding and is influenced by individual (e.g. age, body condition, timing of weaning) and environmental factors (Clutton-Brock *et al.,* 1989; Wade and Schneider, 1992; Gaillard *et al.,* 2000). The cost of reproduction varies but can reduce survival if attempted during periods with low resource availability in seasonal environments (Boyce, 1979; Tavecchia *et al.,* 2005).

Delayed implantation may have evolved in response to seasonal variation in resource availability (Sandell, 1990; Ferguson *et al.,* 1996) allowing females to align reproductive investment with environmental conditions (Stearns, 1989; Murphy, 2012; Renfree and Fenelon, 2017). If a female fails to gain sufficient energetic reserves required for reproduction, termination of pregnancy (e.g. failure to implant) can occur without significant energetic cost (Lake *et al.,* 2008; Robbins *et al.,* 2012a, 2012b). Reproduction requires significant energetic investment and complete litter loss can negatively affect lifetime reproductive success in species that allocate significant energetic reserves to reproduction (Wade and Schneider, 1992). Although delayed implantation may have evolved in response to environmental variability in seasonal environments, evidence supporting this hypothesis is limited (Sandell, 1990; Ferguson *et al.,* 1996; Thom *et al.,* 2004). Alternative hypotheses to explain delayed implantation include: timing of mating with resource availability (mate choice hypothesis) and a vestigial trait that has no current evolutionary advantage (Sandell, 1990; Renfree and Fenelon, 2017). Increasing our understanding of environmental variability on reproductive dynamics for species with delayed implantation is needed especially where climate change is altering ecosystems.

Polar bears (*Ursus maritimus*) have obligate delayed implantation with mating occurring on the sea ice between March and June and implantation occurring in September/October (Ramsay and Stirling, 1986; Ramsay and Stirling, 1988; Derocher *et al.,* 1992). Age of first reproduction in female polar bears typically ranges from 5 to 7 years (Ramsay and Stirling, 1988; Taylor *et al.,* 2008) with lower reproductive output for bears >20 years old related to senescence (Ramsay and Stirling, 1988; Derocher and Stirling, 1994; Naciri *et al.,* 2022), however bears as old as 27 years have been observed with cubs (Derocher *et al.,* 1992). Although litter sizes typically vary from 1 to 3, twins are most common (Ramsay and Stirling, 1988).

Polar bears in Hudson Bay inhabit a seasonal ice area where the melting of sea ice forces all bears onshore during summer until it reforms in November/December (Stirling *et al.,* 1977). High site fidelity results in most bears returning to familiar onshore areas where they rely largely on stored energy reserves through the ice-free period (Derocher and Stirling, 1990; Stirling *et al.,* 2004). While most bears return to the sea ice after 3–5 months onshore, pregnant females enter maternity dens in August through October and fast for up to 8 months from arrival onshore to returning to the sea ice. Gestation and early lactation occur over winter until den emergence in late February/March (Jonkel *et al.,* 1972; Ramsay and Andriashek, 1986; Lunn *et al.,* 2004). Females lose up to 43% of their body mass during this time, of which 93% is derived from fat stores (Atkinson and Ramsay, 1995). These energetic stores are accumulated primarily during the hyperphagic spring period where bears prey upon ringed seals (*Pusa hispida*) and bearded seals (*Erignathus barbatus*) (Stirling and Archibald, 1977; Thiemann *et al.,* 2008; Johnson *et al.,* 2019). Body fat reserves need to comprise 34% of a female polar bear’s body mass for reproduction (Robbins *et al.,* 2012b). While some foraging on terrestrial resources occurs (Russell, 1975; Derocher *et al.,* 1993b) the energetic gain is negligible (Hobson *et al.,* 2009; Pilfold *et al.,* 2016; Pagano *et al.,* 2024). Thus, the energy required for successful reproduction for female polar bears in Hudson Bay is obtained before coming ashore and must be sufficient to complete the capital breeding state that includes gestation and early lactation before a transition to an income breeding state after returning to the sea ice in spring with 3-month-old cubs.

Pregnancy rates in the Western Hudson Bay (WH) polar bear subpopulation were high and ranged from 82 to 100% between 1982 and 1990 (Derocher *et al.,* 1992) but may be affected by changing environmental conditions. For example, there has been an increase in the ice-free period in WH (Gagnon and Gough, 2005; Stern and Laidre, 2016) characterized as a regime shift (Hochheim *et al.,* 2010; Scott and Marshall, 2010), resulting in a decline in polar bear body condition and abundance (Stirling *et al.,* 1999; Regehr *et al.,* 2007; Lunn *et al.,* 2016; Sciullo *et al.,* 2016; Johnson *et al.,* 2020), and an increase in the interbirth interval of successful reproduction due to a delay in weaning of offspring (Ramsay and Stirling, 1988). The proportion of independent yearlings was 81% before 1980 and 34% from 1980 to 1992 and then declined further to 15–20% (Derocher and Stirling, 1995; Stirling *et al.,* 1999). The reason for the reduction in independent yearlings is unknown but was postulated to be related to density-dependent or environmental factors (Derocher and Stirling, 1992). Thus, it is unknown if increases in the ice-free period length and declines in body condition have affected pregnancy rates or the timing of weaning in WH.

The objectives of this study were to investigate long-term trends in pregnancy rates for WH polar bears in relation to body mass, age and sea ice conditions and explore if reproductive status can inform the timing of weaning. We define pregnancy as the implantation stage because this stage is associated with elevated progesterone levels. We predicted that pregnancy rates would be lower over time reflecting changing sea ice conditions and prey availability. We also predicted lower pregnancy rates in older bears due to reproductive senescence. We predicted that heavier bears would be more likely to be pregnant than lighter bears. We hypothesize that adult females who wean offspring as yearlings are able to mate and become pregnant the same year, thus reducing their interbirth interval.

## Materials and Methods

**Figure 1:**
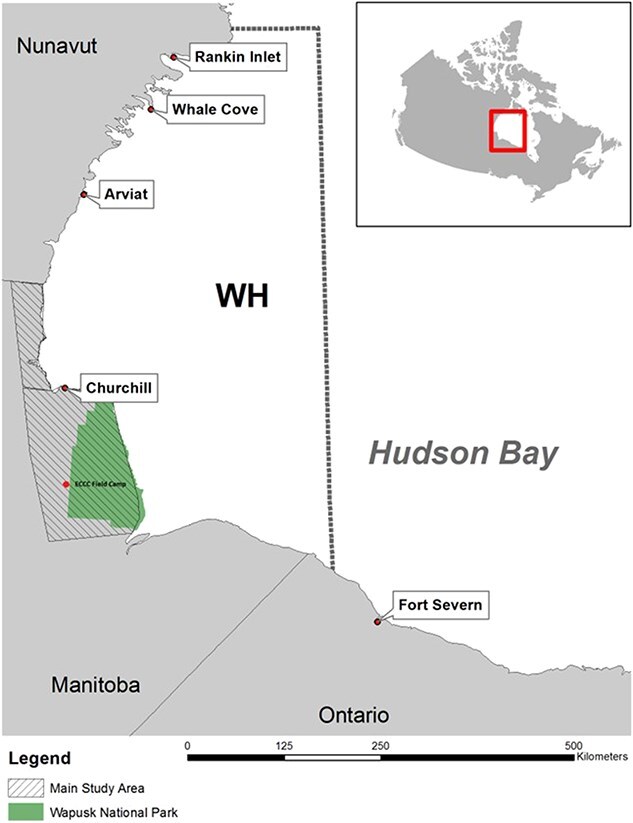
The study area in which free-ranging polar bears were sampled from 1986 to 2021 in northeastern Manitoba, Canada. WH represents the Western Hudson Bay subpopulation boundary

We captured free-ranging polar bears onshore in northeastern Manitoba, Canada, from the Nelson River to the Nunavut border (Fig. 1) using standard chemical immobilization techniques (Stirling *et al.,* 1989). Fieldwork occurred during the ice-free period in late summer to early autumn. Additional fieldwork occurred in late February and March when females emerging from maternity dens were captured as they migrated back to the sea ice. Spring observations were used to assist in confirmation of pregnancy and whole-litter loss.

We collected blood from immobilized bears from 1991 to 2021 with the exceptions of 2002, 2011–14 and 2020. Blood was collected from the femoral or jugular vein into serum separator vacutainers (Becton, Dickinson and Co., Franklin Lakes, NJ), centrifuged for 10–20 min and the serum decanted and stored frozen at −80°C until analysis. Serum progesterone levels were assessed using radioimmunoassay by Prairie Diagnostic Services Inc. at the University of Saskatchewan (Saskatoon, SK). Females with progesterone levels ≥2.5 ng/ml during the late summer/autumn were considered pregnant and those below that level as non-pregnant (Derocher *et al.,* 1992). We analysed serum samples for females that were independent and ≥3 years old (a small number of 2-year-olds were analysed), and for all adult females (≥5 years of age) even if accompanied by offspring.

We estimated body mass (in kilogrammes) for each bear captured from standardized measures of straight-line body length (in centimetres) and axillary girth (in centimetres) using separate equations for bears captured up to and including 1996 and years after 1996 (Thiemann *et al.,* 2011). Mass was standardized to September 1 based on female mass loss rate of 0.9 kg/day (Derocher and Stirling, 1995; Pagano *et al.,* 2024) because females were captured primarily in late August through early October. A subjective index of body fat (1–5) was also recorded, where 1 and 5 represented skinny and obese bears, respectively (Stirling *et al.,* 2008). A vestigial premolar tooth was removed for subsequent age determination from counts of cementum annuli (Calvert and Ramsay, 1998). Bears captured as known-age cubs-of-the-year (cubs) or dependent yearlings were aged based on tooth eruption patterns. Research was approved annually by the Environment and Climate Change Canada Western and Northern Animal Care Committee and conducted under wildlife research permits issued by the Province of Manitoba and Parks Canada Agency.

Sea ice data was derived from SSM/I SSMIS Nimbus satellite sensors with a spatial resolution of 25 × 25 km from 1980 to 2021 (Cavalieri *et al.,* 1996) and restricted to the WH subpopulation boundary (Lunn *et al.,* 2016). Sea ice covariates we considered were: (i) retreat date, defined as the ordinal date when sea ice extent within the WH subpopulation boundary was ≤10% of the total area and where sea ice was defined as ≥30% ice concentration for an individual grid cell (Cherry *et al.,* 2013; McGeachy *et al.,* 2024b), (ii) breakup date, where the mean ice concentration in WH dropped <50% and stayed <50% for three consecutive days (Lunn *et al.,* 2016), (iii) number of ice-covered days (ice_days) calculated as the number of days in the calendar year – the ordinal date sea ice extent returned to 10% of the area in WH (Ice advance) + the ordinal date of the ice retreat date the following year and 4) a lag effect in ice_days where the number of ice-covered days the previous year was considered (ice_lag). Ice metric 3 represented the number of ice-covered days experienced before coming ashore while the lag represented the number of ice-covered days from the previous year. We assessed temporal trends in sea ice metrics with both linear regression and broken-stick regression (R package segmented) and evaluated model fit using Bayesian information criteria (BIC) (Tiwari *et al.,* 2005).

### Statistical analysis

To assess temporal trends in the pregnancy rate we used a linear regression where year was the predictor and the pregnancy rate was the response variable. We built one model using data from 1991 to 2021 and a second including previously published data from Derocher *et al.* (1992) (1982–90) combined with the 1991–2021 dataset. We compared the pregnancy rate between 1982 and 1990 with the pregnancy rate from 1991 to 2021 using a two-sample proportion test. To assess changes in first breeding (referred to as such because we cannot confirm parturition) we investigated changes in pregnancy rate specifically for 4-year-old females between 1982 and 1990 (previously published) and 1991–2021 using a two-sample proportion test. Progesterone values were available from 179 individuals collected on 210 occasions from 1986 to 1990 (Derocher *et al.,* 1992). Linear regression was used to assess changes in mass of pregnant or non-pregnant females over time (1986–2021 and separately for 1991–2021) and assess the relationship between mass of pregnant females and sea ice conditions. In these models’ mass was the response variable. We also assessed linear and non-linear relationships to assess the effects of age on mass for both pregnant and non-pregnant solitary females. We assessed normality using a residual plot against the fitted values of the linear model, a histogram of the residuals and QQ plots against the fitted values. If residuals were non-normal and transformations did improve normality, we used non-parametric tests.

We used a one-way ANOVA to test differences in mass between females ≥5 years old captured with yearlings, with cubs, pregnant or non-pregnant females between 1991 and 2021. Comparison of mass between females based on reproductive status was restricted to 1991–2021 because earlier data did not evaluate progesterone levels for females with cubs. A Tukey’s test was used to compare differences between groups. We estimated age of first breeding similar to Derocher *et al.* (1992) by estimating the proportion of bears that bred <5 years old from progesterone levels and assumed all remaining females bred at 5 years.

We used a generalized linear model with a binomial distribution to investigate the influence of age, body condition (mass or fat index) and sea ice conditions on pregnancy status (1 = pregnant, 0 = non-pregnant) using data from 1986 to 1990 (published in Derocher *et al.,* 1992, data from 1982 to 1985 was not available) combined with data from 1991 to 2021 (this study). The individual covariates used were age (years), quadratic term for age (age + age^2^), mass (in kilogrammes) and fat index.

We used backward selection for the generalized linear model and removed one non-significant covariate with each iteration until all covariates were significant. We assessed model fit using McFadden’s *R*^2^ and used Akaike information criteria corrected for small sample size (AICc) for model selection of the candidate set with a ΔAICc of ≥2 indicating superior fit (Burnham and Anderson, 2002). With model selection uncertainty, we chose the most parsimonious model. We used Pearson correlation to identify highly correlated (>0.6) covariates and evaluated each correlated covariate independently in the model including only the top fitting correlated covariate in the full model. Significance was assessed at *P* ≤ 0.05.

To determine the timing of weaning of yearlings (ca. 1.8 years old) that were captured alone in the autumn, we used capture histories to assess the status of mothers during the same season and assumed females were solitary if they bred and remained so afterwards. Females that weaned yearlings were considered pregnant if they were captured in the same year as their independent yearling and had progesterone levels ≥2.5 ng/ml, captured in year *t* + 1 with cubs or captured in year *t* + 2 with yearlings. Females where reproductive status in year *t* could not be determined were not included. All statistics were performed in R (R Development Core Team, 2021) in version 4.0.4.

## Results

Serum progesterone levels were determined for 541 samples collected from 441 individuals from 1991 to 2021. The mean age of sampled females was 11.8 years (SE = 0.1, range: 2–31 years). Subadults (2–4 years old) represented 17% (*n* = 92) and adults 83% (*n* = 449) of the sample. The majority of the samples from adult bears were from solitary females (63%, *n* = 283), followed by females with cubs (26%, *n* = 116) and females with yearlings (11%, *n* = 48). Two samples were not used in the analysis, one adult female accompanied by a 2-year-old and one female accompanied by both a yearling and a cub. Using microsatellites (Malenfant *et al.,* 2016), we confirmed the cub was adopted and not related to the mother (did not share 1 allele at each loci) and the yearling was the biological offspring (shared 1 allele at each loci).

### Sea ice

**Figure 2:**
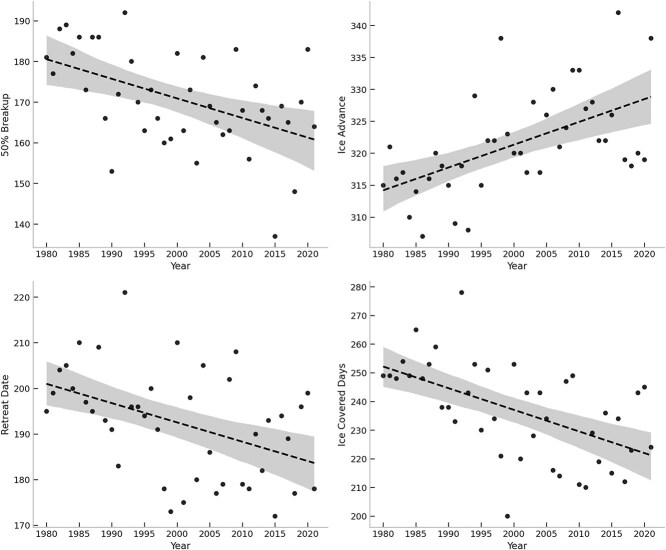
Linear regression for four ice metrics assessed in Western Hudson Bay; 50% breakup date (top left), ice advance (top right), retreat date (bottom left) and ice-covered days (bottom right); dashed line represents linear regression

Linear regression fit trends in sea ice metrics better than broken-stick regression for all ice metrics; 50% breakup (ΔBIC 4.00), retreat date (ΔBIC 6.29), ice advance (ΔBIC 5.05) and ice_days (ΔBIC 3.74). We found significant declines in the breakup date (*F*_1 40_ = 13.10, *P* < 0.001), retreat date (*F*_1 40_ = 9.45, *P* < 0.001), ice_days (*F*_1 40_ = 17.28, *P* < 0.0001) and a significant increase in ice advance (*F*_1 40_ = 17.64, *P* < 0.001) (Fig. 2).

### Progesterone levels

The mean serum progesterone level was 2.69 (SE = 0.08, range: 0.00–20.49 ng/ml). No female accompanied by offspring had progesterone levels ≥2.5 ng/ml (max = 1.09 ng/ml). Although no female accompanied by offspring was pregnant, progesterone levels of females accompanied by yearlings (mean = 0.33, SE = 0.04, range: 0–2.02 ng/ml) were significantly higher (*t*_163_ = −3.58, *P* < 0.001) than females accompanied by cubs (mean = 0.08, SE = 0.01, range: 0–0.70 ng/ml).

**Figure 3:**
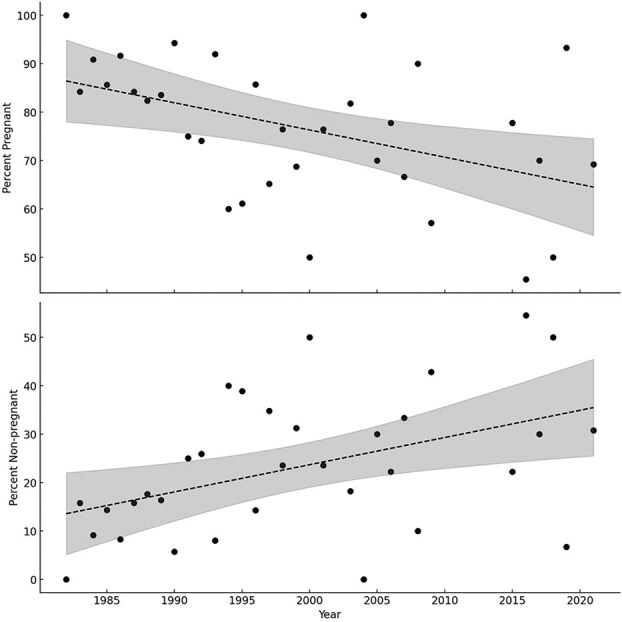
Percent pregnant (progesterone ≥2.5 ng/ml, top) and non-pregnant (progesterone <2.5 ng/ml, bottom) solitary female polar bears ≥4 years old captured onshore in the Western Hudson Bay subpopulation northeastern Manitoba, from 1982 to 2021. Data from 1982 to 1990 was from Derocher *et al.* (1992). Dashed line represents significant linear regression (*P* < 0.01), shaded area represents 95% CI

Pregnancy rates for females ≥4 years old declined significantly (*F*_1 31_ = 7.89, *P* < 0.01, *R*^2^ = 0.20, slope = −0.56) from 1982 to 2021 (Fig. 3), however, the trend was non-significant from 1991 to 2021 (*F*_1 22_ = 0.33, *P* = 0.57). The pooled pregnancy rate from 1991 to 2021 was 72.5% (Table 1, range: 45.5–100%) and significantly lower (*X*^2^ = 11.89, *df* = 1, *P* < 0.001) compared to 84.8% (range: 82.4–100%) from 1982 to 1990 (Derocher *et al.,* 1992). Interannual variation in pregnancy rate (i.e. coefficient of variation) between periods increased almost 3-fold from 6.7% in 1982–90 to 19.6% in 1991–2021 (excluding 2010 with *n* = 1). The pregnancy rate of 4-year-old females was significantly lower (*X*^2^ = 6.13, *df* = 1, *P* = 0.01) in 1991–2021 (54.9%, *n* = 51) compared to 1982–90 (82.0, *n* = 39) reported by Derocher *et al.* (1992). The age of first breeding was estimated as 4.5 years old. Age-specific pregnancy rates increased with age and declined after 23 years old (Table 2). No 2- or 3-year-old females were pregnant. The oldest pregnant female was 30 years old, and the oldest female accompanied by cubs was 28 years.

**Table 2:** Age-specific pregnancy rate (presented as percent pregnant) of solitary, free-ranging female polar bears captured in autumn, from 1991 to 2021 (excluding 2002, 2011–14 and 2020) from the Western Hudson Bay subpopulation in Canada

Age	Percent pregnant	*n*
2	0	4
3	0	37
4	54.9	51
5	86.8	38
6	87.5	24
7	75.0	20
8	79.2	24
9	78.6	14
10	80.0	10
11	81.3	16
12	91.7	12
13	58.3	12
14	81.8	11
15	91.7	12
16	75.0	4
17	100.0	4
18	90.9	11
19	60.0	5
20	100.0	3
21	100.0	5
22	100.0	4
23	100.0	7
24	55.6	9
25	37.5	8
26	50.0	8
27	14.3	7
28	50.0	6
29	00	4
30	25.0	4
31	0.0	1

Pregnancy was determined from serum progesterone concentrations, where values ≥2.5 ng/ml were considered pregnant.

**Table 1:** Pregnancy rate (presented as percent pregnant) for solitary female polar bears ≥4 years old based on serum progesterone concentrations collected from free-ranging polar bears in autumn in the Western Hudson Bay subpopulation from 1991 to 2021 (excluding 2002, 2011–14 and 2020)

Year	Percent pregnant	*n*
1991	75.0	24
1992	74.1	27
1993	92.0	25
1994	60.0	15
1995	61.1	18
1996	85.7	14
1997	65.2	23
1998	76.5	17
1999	68.8	16
2000	50.0	8
2001	76.5	17
2003	81.8	11
2004	100.0	5
2005	70.0	10
2006	77.8	9
2007	66.7	3
2008	90.0	10
2009	57.1	7
2010	0.0	1
2015	77.8	9
2016	45.5	11
2017	70.0	10
2018	50.0	16
2019	93.3	15
2021	69.2	13
Overall	72.5	334

Age was not normally distributed, and transformations did not improve normality. A Mann–Kendall test was used to assess trends in age for pregnant and non-pregnant females (Mann, 1945; Kendall, 1975). There was no temporal trend in the age of pregnant females (Mann–Kendall, *z* = 0.357, tau = 0.049, *P* = 0.721) or non-pregnant females (*z* = −1.089, tau = −0.143, *P* = 0.276).

### Mass

**Figure 4:**
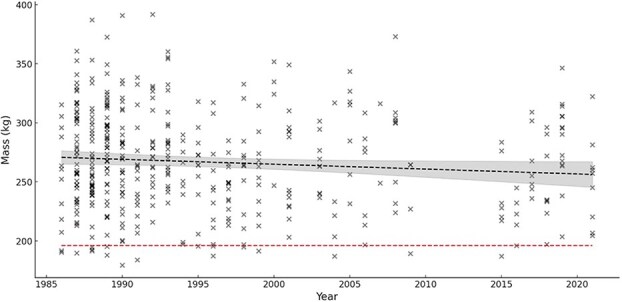
Mass (in kilogrammes) of pregnant female polar bears standardized to September 1 captured onshore in the Western Hudson Bay subpopulation, 1986–2021. Lower horizontal dashed line represents the minimum mass of a confirmed pregnant female. Black dashed line is a linear regression (*P* = 0.04). Shaded area represents 95% CI

**Figure 5:**
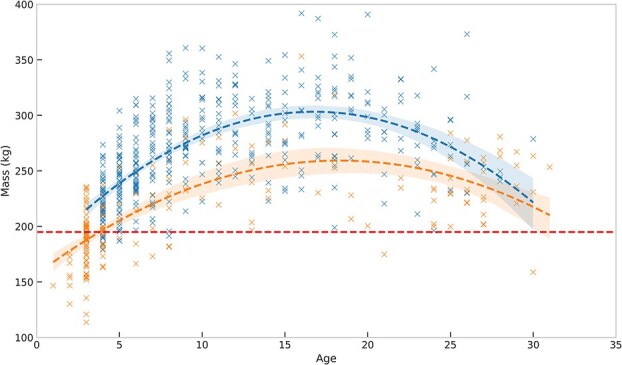
Relationship between age and mass (in kilogrammes, standardized to September 1) for female polar bears captured onshore in the Western Hudson Bay subpopulation, in 1986 to 2021 for pregnant (≥2.5 ng/ml progesterone, blue x) and non-pregnant (<2.5 ng/ml progesterone, orange x) individuals. Dashed line represents non-linear relationship for pregnant (*R*^2^ = 0.40) and non-pregnant (*R*^2^ = 0.45) females. Red dashed line represents the lightest confirmed pregnant female (195 kg) and shaded area represents 95% CI for the fitted model

**Figure 6:**
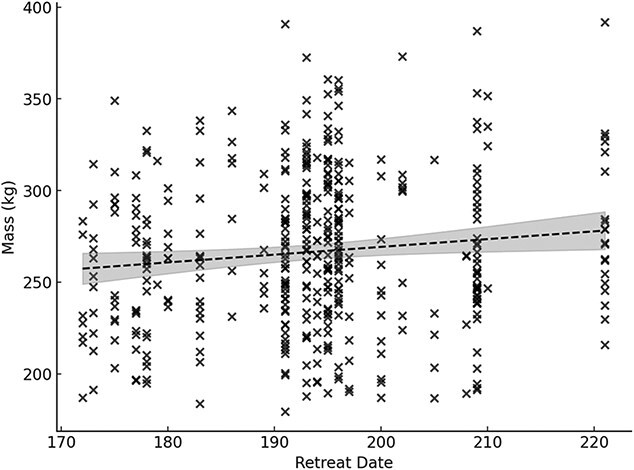
Mass (in kilogrammes) of pregnant (serum progesterone ≥2.5 ng/ml) polar bears standardized to September 1 captured onshore in the Western Hudson Bay subpopulation, for sampling years 1986–2021 in relation to remnant ice retreat date in Western Hudson Bay. Error bars are one standard error of the mean. Dashed line represents significant linear regression (*P* = 0.02) and shaded area is the 95% CI for fitted model

There was no temporal trend in mass of pregnant females between 1991 and 2021 (linear regression, *F*_1, 240_ = 1.03, *P* = 0.31); however, there was a significant decline between 1986 and 2021 (linear regression *F*_1, 422_ = 4.06, *P* = 0.04, *R*^2^ = 0.01) (Fig. 4). For this model the slope was −0.41 indicating, on average, mass decreases by 0.41 kg/year. Mass was influenced by age with a non-linear relationship that explained 40% of the variation (*R*^2^ = 0.40, Fig. 5). There was no temporal trend in non-pregnant female mass between 1991 and 2021 (linear regression, *F*_1, 131_ = 2.51, *P* = 0.12) or between 1986 and 2021 (linear regression, *F*_1, 195_ = 1.04, *P* = 0.31). There was no significant difference (*t*_80_ = 1.99, *P* = 0.08) in mass of 4-year-old solitary females from 1986 to 1990 (mean = 219 kg, SE = 3.6, *n* = 31, range: 179–261 kg) compared to 1991 to 2021 (mean = 209 kg, SE = 3.5, *n* = 51, range: 163–273 kg). The lightest female that had a progesterone level ≥2.5 ng/ml was a 4-year-old with a mass of 179 kg adjusted to September 1 (169 kg at capture). The lightest female that had progesterone ≥2.5 ng/ml and later confirmed pregnant by a capture the following year with cubs was an 8-year-old that had an estimated mass of 195 kg adjusted to September 1 (196 kg at capture). This female was captured alone in autumn 1996 and was subsequently captured in autumn 1997 with two male cubs, both of which were later recaptured as adults. Sixteen females had progesterone levels ≥2.5 ng/ml but were lighter than the minimum confirmed pregnant female (195 kg) and 94% of them were ≤5 years old. Pregnant females were significantly heavier in years with later ice retreat dates (linear regression, *F*_1, 422_ = 5.79, *P* = 0.02, *R*^2^ = 0.01) (Fig. 6). The slope of the regression was 0.42 indicating for every 1 day the retreat date increased (i.e. a longer ice period) pregnant females on average were 0.42 kg heavier; however, this relationship only explained 1% of the variation.

**Figure 7:**
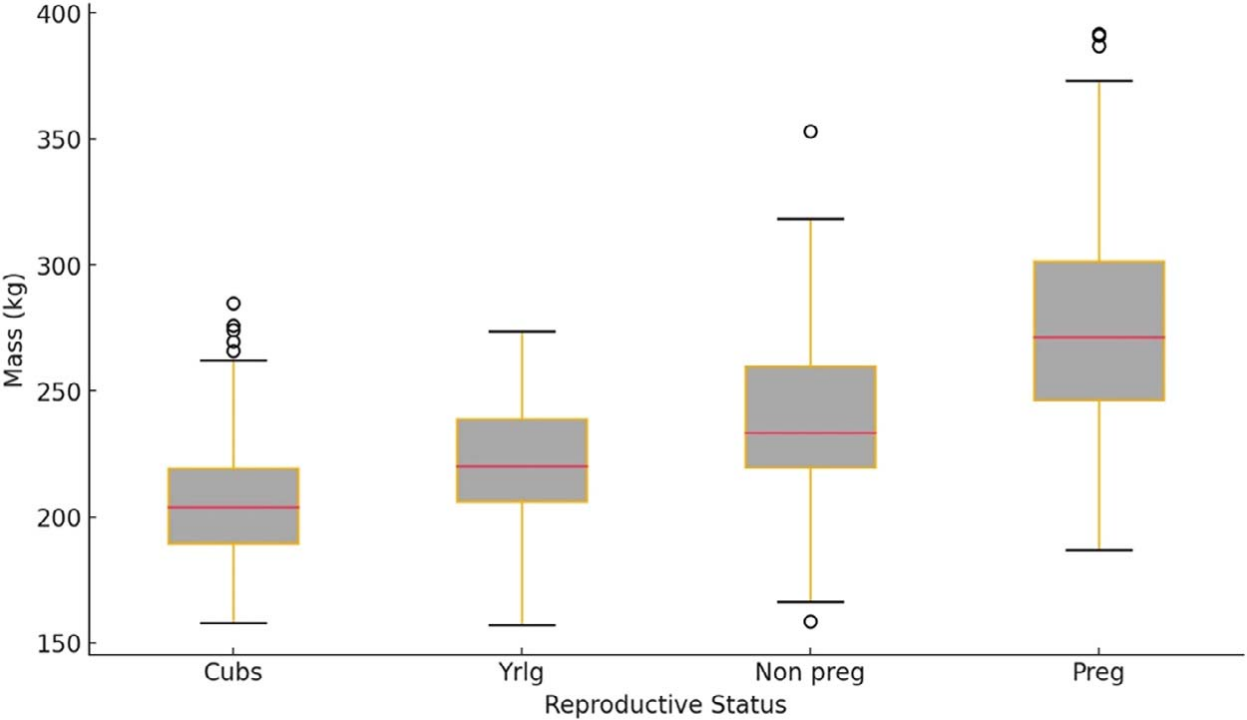
Box plot of adult female (>4 years old) polar bear mass (in kilogrammes) standardized to September 1 for females captured accompanied by cubs-of-the-year (Cubs), yearlings (Yrlg), non-pregnant (Non preg, <2.5 ng/ml progesterone) and pregnant (Preg, ≥2.5 ng/ml progesterone) and captured onshore in the Western Hudson Bay subpopulation in 1991–2021. Horizontal line inside the box represents the median and the box represents the lower (25th) and upper (75th) quartile. Whiskers represent the range and points represent outliers

There was a significant difference in mass for females ≥5 years old between solitary pregnant females, solitary non-pregnant females and females accompanied by offspring (*F*_3, 444_ = 92.05, *P* < 0.001, Fig. 7). Pregnant females were significantly heavier than non-pregnant females (Tukey’s test, *P* < 0.001), female with yearlings (Tukey’s test, *P* < 0.001) and females with cubs (Tukey’s test, *P* < 0.001). Non-pregnant females were significantly heavier than females with yearlings (Tukey’s test, *P* < 0.01) and females with cubs (Tukey’s test, *P* < 0.001). Females with yearlings were not significantly heavier than females with cubs (Tukey’s test, *P* = 0.32).

Of the 88 bears that were confirmed pregnant, only one bear had progesterone levels <2.5 ng/ml. This bear had a progesterone level of 0.40 ng/ml and was captured on 20 July 1996, which may have been before implantation and elevated progesterone. The second lowest progesterone level of a confirmed pregnant female was 2.53 ng/ml by a female captured on 24 August 1995. Of 116 females presumed pregnant, 55% were observed with cubs the following year, 21% (*n* = 24) were observed with yearlings and 24% (*n* = 28) were captured alone the following year. For females observed with cubs the following year, 16% (10/64) were with cubs in March and subsequently captured alone in September. Thus, the proportion of pregnant females recaptured alone the following year increased to 28% if these are included. Of the 10 females that were pregnant in autumn, with cubs the following March, and then alone the following autumn, 50% were 10–18 years old and 50% were 6–9 years old. The mean autumn mass of these 10 females was 275 kg (SE = 14.7, range: 237–387 kg). Twenty pregnant females were physically recaptured alone the following year. Of these recaptures 70% (*n* = 14) were classified as pregnant.

### Generalized linear model

**Figure 8:**
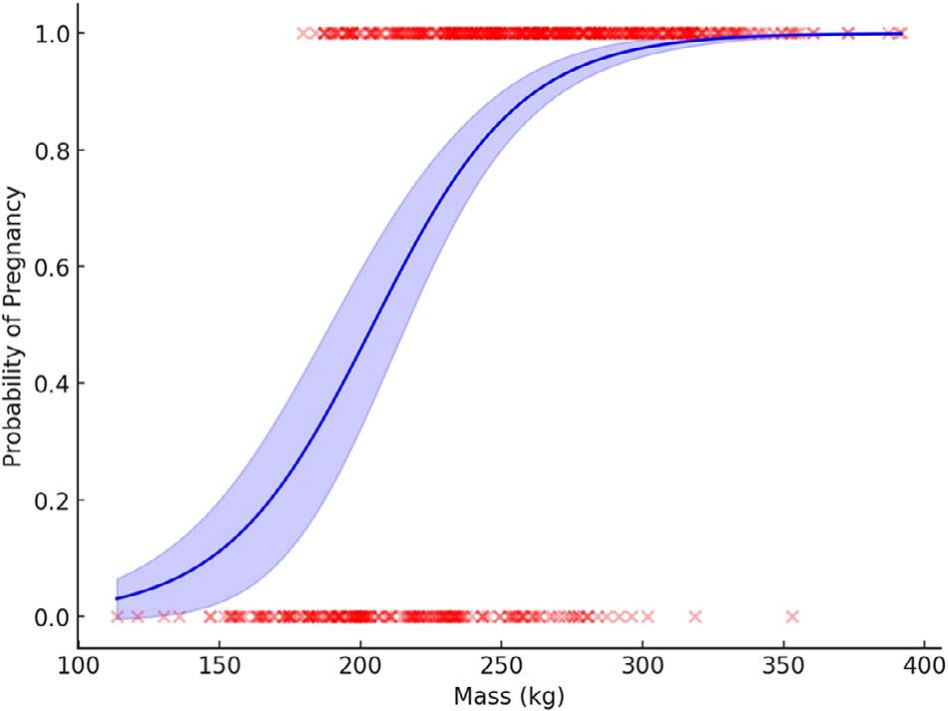
Effects of mass (in kilogrammes) on the probability of pregnancy from the top generalized linear model for female polar bears ≥4 years old, captured onshore in the Western Hudson Bay subpopulation in 1986–2021. Shaded area represents 95% CI

**Figure 9:**
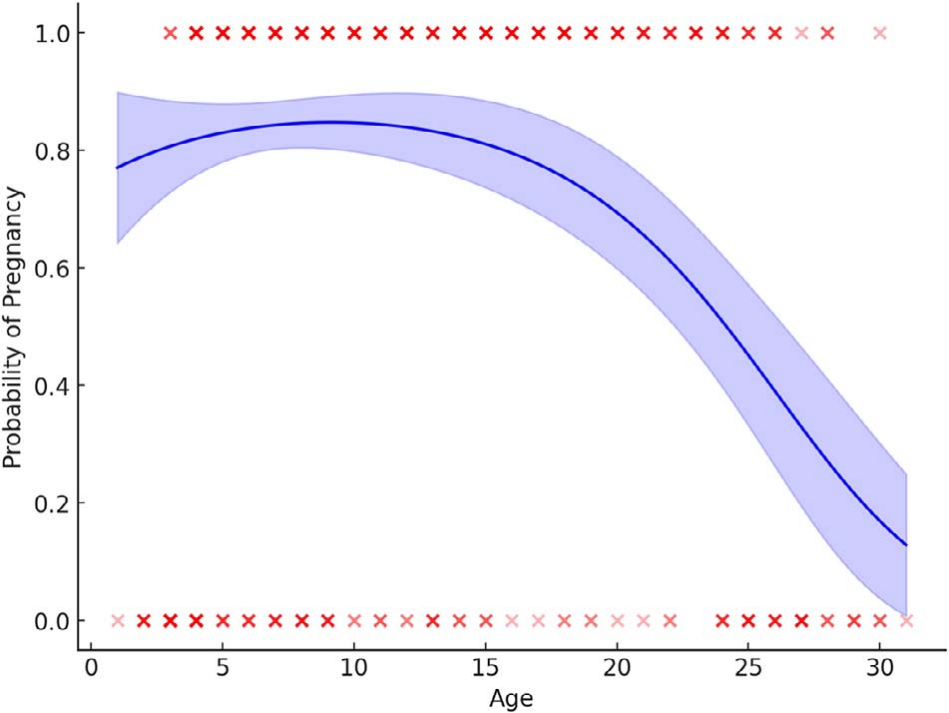
Effects of age (years) on the probability of pregnancy from the top generalized linear model for female polar bears ≥4 years old, captured onshore in the Western Hudson Bay subpopulation in 1986–2021. Shaded area represents 95% CI

The generalized linear model with a binomial distribution included 622 samples from solitary females collected from 480 individuals ranging in age from 1 to 31 years old from 1986 to 2021. Of these, 424 (68%) were pregnant and 198 (32%) were classified as non-pregnant. Of the 480 solitary bears, 373 (78%) were captured once, 79 (16%) were captured twice and 28 (6%) were captured more than twice (note this refers only to females captured as solitary for the GLM, bears may have been recaptured with cubs or yearlings and were excluded). Retreat date, breakup date and the number of ice-covered days were highly correlated as was mass and fat index. Ice_days and mass were included in the full model because they had the best model fit compared to the other correlated covariates. The top model included the covariates mass and age^2^ (McFadden’s *R*^2^ = 0.33: Tables 3 and 4). Mass was a strong predictor of pregnancy with a 3.88% increase in the probability of being pregnant for every 1 kg increase in mass. Heavier bears were more likely to be pregnant with the inflection point in the model occurring at 214 kg (Figs 5 and 8). Pregnancy probability increased slightly with age and then declined for older bears with a non-linear relationship (Table 4, Figs 5 and 9).

**Table 3:** Generalized linear model results using backward selection to determine factors that influence pregnancy rates in polar bears in the Western Hudson Bay subpopulation from 1986 to 2021 ranked by AICc

Model	Covariates	AICc	k	Deviance	ΔAICc	Model weight
4	Mass + Age^2^	529.379	4	521.282	0.000	0.401
3	Mass + Year + Age^2^	530.043	5	519.906	0.664	0.288
2	Mass + Year + Age^2^ + Ice_days	530.619	6	518.436	1.240	0.216
1	Mass + Year + Age^2^ + Ice_days + Ice_lag	532.258	7	518.022	2.879	0.095

**Table 4:** Generalized linear regression coefficients from the most parsimonious model influencing the probability of pregnancy in female polar bears in Western Hudson Bay from 1986 to 2021

Coefficients	Estimate	SE	Significance
Intercept	−8.410582	0.801039	<0.0001
Mass	0.038087	0.004126	<0.0001
Age	0.139026	0.078429	0.076
Age^2^	−0.007600	0.002546	<0.001

From 1980 to 2021, 23 solitary females were captured in the same year as their weaned independent yearlings and 91% (21) were pregnant. The mean age of these pregnant adult females was 15 years (SE = 0.4, range: 7–21 years). From 1993 to 2021, 9% of yearlings were independent and ranged from 0% in 8 different years to 25% in 2019.

## Discussion

Assessing changes in pregnancy rates improves our understanding of individual and environmental effects on polar bear reproductive dynamics that influence age structure, recruitment and population growth. Using a long-term dataset of serum progesterone levels in female polar bears, we documented declining pregnancy rates and increased interannual variation over 4 decades and identified individual covariates that were associated with the probability a solitary female was pregnant. Further, age of first breeding was delayed over time and coincided with a longer ice-free period.

The individual factors that most influenced pregnancy rates were mass and age^2^. Mass was a better predictor of pregnancy than fat index possibly because of the coarse and subjective nature of the body condition data. Heavier bears were more likely to be pregnant suggesting that obtaining sufficient mass before coming ashore was critical for successful reproduction. Our findings support past research highlighting the importance of age and mass for reproductive success (Atkinson and Ramsay, 1995; Derocher and Stirling, 1995; Robbins *et al.,* 2012a, 2012b). Our minimum estimated mass of a confirmed pregnant female was 196 kg, which is similar to 189 kg from Derocher *et al.* (1992). The 196-kg female’s cubs were both recaptured as adults and indicate a possible approximate minimum mass females need to produce cubs and still possess sufficient energetic reserves to complete the full capital breeding reproductive cycle of gestation, early lactation and migration back to the sea ice before transitioning to an income breeding state. It is unknown, however, if females that were lighter and pregnant also successfully completed the capital breeding cycle. Non-pregnant solitary females may have failed to implant due to poor body condition or perhaps lost cubs after the mating season prior to coming ashore. The energy stores of solitary females declined between 1985 and 2018 in western Hudson Bay (Johnson *et al.,* 2020). We found a decline in mass of pregnant but not non-pregnant adult females from 1986 to 2021, a decline in the pregnancy rate and that pregnant females were heavier compared to non-pregnant females. Johnson *et al.* (2020) suggested that the inclusion of more non-pregnant adult females in the solitary adult female class in their study may have contributed to declining energy stores of solitary females and our study supports this.

The non-linear term for age (age + age^2^) was a strong predictor of polar bear pregnancy rates and improved the model fit compared to the linear relationship with age, supporting reproductive senescence in polar bears. Polar bear reproductive senescence is challenging to document due to low samples sizes at the upper end of an age structure, but it has been suggested (e.g. Ramsay and Stirling, 1988; Derocher *et al.,* 1992; Derocher and Stirling, 1994; Folio *et al.,* 2019; Naciri *et al.,* 2022). Although pregnancy rates declined for bears in their mid-20s, similar to findings of Derocher *et al.* (1992), bears as old as 30 years were pregnant. The costs of reproduction are high during gestation and lactation (Clutton-Brock *et al.,* 1989) but pregnancy is energetically inexpensive for polar bears at the implantation stage even if they cannot complete the full reproductive cycle (Derocher *et al.,* 1992).

Age of first reproduction is an important aspect of life history strategies that can influence population dynamics (Cole, 1954; Roff, 1992) and our results suggested a delay in first breeding (we could not confirm parturition for all pregnant females) possibly related to environmental change. Age at first reproduction tends to increase when resources are limited for large vertebrates (Eberhardt, 2002). The ice-free period has increased in WH (Scott and Marshall, 2010; Stern and Laidre, 2016) and may have influenced pregnancy rates of 3-year-old bears which declined from 8.9 (Derocher *et al.,* 1992) to 0% (this study). Similarly, 82.0% of 4-year-olds were pregnant in 1982–90 (Derocher *et al.,* 1992) compared to only 54.9% in 1991–2021 during our study. The mean age of first breeding between 1982 and 1990 was estimated at 4.1 years under the assumption all 5-year-olds breed (Derocher *et al.,* 1992). If we assume all 5-year-olds breed to compare the estimate with Derocher *et al.* (1992), then the average age of first breeding is now 4.5 years. The increase in age of first breeding could be higher if not all females breed at 5 years old. While age of first reproduction for polar bears varies from 4 to 7 years across the circumpolar Arctic in relation to environmental conditions (Ramsay and Stirling, 1988; Taylor *et al.,* 2008), knowledge of variation in age of first breeding within a subpopulation in relation to environmental change are limited (Ramsay and Stirling, 1988; Derocher and Stirling, 1995; Medill *et al.,* 2010).

We postulate that a delay in first breeding may have delayed senescence in our population. Derocher *et al.* (1992) reported low pregnancy rates for females in their early 20s but our study estimated 100% pregnancy rates from serum progesterone levels for females in their early 20s until they reached 24 years old. However, we had small sample sizes for older bears. We suggest that females may have delayed reproduction in response to a longer ice-free season. Attaining 97% of their asymptotic body length was suggested as necessary for primiparity in polar bears (Derocher  and Stirling, 1998b), which is consistent with trade-offs associated with reproduction (Stearns, 1989). Increasing the somatic growth period to reach primiparity body size in our study population may have delayed maturation supporting the suggestion that food availability is an important environmental factor influencing reproduction (Wade and Schneider, 1992; Lunn *et al.,* 1994). An older age of primiparity for black bears (*Ursus americanus*) was found in areas with lower food availability (Wightman *et al.,* 2022). Primiparity in brown bears (*Ursus arctos*) was thought to be related to social cues with delays associated with higher home range overlap between mother and daughters possibly related to inbreeding avoidance (Støen *et al.,* 2006). Polar bears in our study area have large home ranges and occur at low density (McCall *et al.,* 2015) and thus social cues are unlikely to influence primiparity. Thus, if our finding of fewer pregnant 4-year-olds results in an increase in the age of primiparity, senescence may be delayed, which is consistent with life history theory (Roff, 1992).

It is important to note that pregnancy does not equal parturition. Derocher *et al.* (1992) found that whole-litter loss accounted for most, but not all, of the females that were presumed pregnant and not subsequently seen with cubs and suggested that foetal absorption, pseudopregnancy and perhaps cub mortality inside dens may also occur. Medill *et al.* (2010) used cementum annuli to determine primiparity in polar bears in WH and found 41% of 4-year-olds gave birth between 1993 and 2006. Thus, our pregnancy rate of 55% for 4-year-old bears suggests ~14% may incur foetal absorption or were pseudopregnant. Most of the pregnant females that were below the minimum mass of a confirmed pregnant female were ≤5 years old and may have lacked sufficient energetic reserves to complete the capital breeding cycle. Pseudopregnancy results in elevated progesterone levels similar to pregnant females (Shimozuru *et al.,* 2013), which we were unable to differentiate. The hormone relaxin present in pregnant females after implantation can distinguish pregnancy from pseudopregnancy in canids (Carlson and Gese, 2007) and felids (de Haas van Dorsser *et al.,* 2006) but this has not been demonstrated in ursids. We acknowledge some bears may have been pseudopregnant but suggest the proportion is likely low because cub loss accounted for the majority of suspected pregnant females subsequently seen without cubs (Derocher *et al.,* 1992). The decline in pregnancy rates we found would be possibly greater in some years if pseudopregnancy rates were high. The decline in pregnancy rates we found is equivalent to an increase in non-pregnant solitary females, which is not confounded by pregnant and pseudopregnant individuals. Nevertheless, the inability to distinguish pregnant and pseudopregnant females should be considered when interpreting the results and quantification of relaxin levels in polar bears may yield new insights.

Some bears we classed as non-pregnant may have lost cubs on the sea ice after the mating season but before coming ashore as suggested by Derocher *et al.* (1992). This is supported by non-pregnant females being heavier than females with offspring suggesting if they lost cubs in the current year, they had sufficient time on the sea ice to accumulate more fat reserves than lactating females before coming ashore. Alternatively, mass loss rates may differ between lactating and non-lactating females (Miller *et al.,* 2022; Archer *et al.,* 2023), which may partially account for mass differences. Polar bear cub mortality rates are high (Derocher and Stirling, 1995; Lunn *et al.,* 2016) and the timing and cause of death of cubs is largely unknown but thought to be related to infanticide (McGeachy *et al.,* 2024a), starvation (Derocher and Stirling, 1996) and wolf predation (Ramsay and Stirling, 1984).

Despite significant changes to sea ice dynamics in western Hudson Bay, we did not find a strong relationship between our sea ice metrics and pregnancy rates. Although no sea ice metric was included in our top generalized linear model, pregnant females were significantly heavier in years with later ice retreat dates. Such heavier pregnant females indicate a link between environmental conditions and reproductive success because heavier bears have heavier cubs and heavier cubs have higher survival (Atkinson and Ramsay, 1995; Derocher and Stirling, 1996, 1998). We found no significant temporal trend in mass from 1991 to 2021 but did find a significant negative trend from 1986 to 2021. The lack of trend since 1991 may be a result of sea ice stability in Hudson Bay in recent decades (Scott and Marshall, 2010; Lunn *et al.,* 2016; Ferguson *et al.,* 2020). A period of ice stability appears to have provided some reprieve from negative impacts of a longer ice-free season (Lunn *et al.,* 2016). Negative impacts of climate warming were documented in WH (Stirling *et al.,* 1999; Regehr *et al.,* 2007) and projected declines in sea ice (Castro de la Guardia *et al.,* 2013; Stroeve *et al.,* 2024) are predicted to have detrimental effects on reproduction and population persistence (Stirling and Derocher, 1993; Derocher *et al.,* 2004; Robbins *et al.,* 2012b; Molnár *et al.,* 2020; Pagano *et al.,* 2024). The absence of a sea ice metric in our top model may be due to the obligate form of delayed implantation in polar bears and their short gestation being energetically inexpensive and thus an aspect of reproduction less likely to be currently affected by climate change. More energetically demanding aspects of reproduction such as lactation (Miller *et al.,* 2022; Archer *et al.,* 2023) are likely to be more sensitive to changing sea ice conditions. It is also possible alternative sea ice metrics we did not assess may have a stronger relationship with pregnancy rates such as sea ice fragmentation (Biddlecombe *et al.,* 2021), the rate of ice decay (Lunn *et al.,* 2016) or the spatial scale at which sea ice is assessed (McGeachy *et al.,* 2024b).

The population dynamics of seals in Hudson Bay in response to sea ice conditions may influence polar bear pregnancy rates. Declining ringed seal abundance may be related to large-scale atmospheric patterns (Young *et al.,* 2015), lower seal pregnancy rates (Stirling, 2005) and reduced body condition (Ferguson *et al.,* 2017). However, the dynamics of ringed seal populations is difficult to quantify and collected intermittently, making direct comparisons between the synchrony in reproductive dynamics between the two species speculative. For example, ringed seal pregnancy rates were 100% in 2003, 2005 and 2006 suggesting high pup availability should occur the following year (Chambellant *et al.,* 2012) but polar bear pregnancy rates for these years (2004, 2006, 2007) varied and were 100, 77.8 and 66.7%, respectively. Currently the relationship between ringed seal and polar bear pregnancy rates is unclear. Ringed seal abundance may be more influential on polar bear reproductive rates. Estimates derived from aerial surveys in western Hudson Bay show high interannual variation in ringed seal abundance estimates with a possible declining or cyclic trend (Young *et al.,* 2015).

Interannual variation in pregnancy rates was higher after 1990 (this study) compared to 1982–90 (Derocher *et al.,* 1992). Some years pregnancy rates were as low as 45.5%. A low pregnancy rate could result in lower cub production and may be contributing to the lower productivity in WH compared to the neighbouring Southern Hudson Bay subpopulation (Atkinson *et al.,* 2022; Northrup *et al.,* 2022). The increase in interannual variation in pregnancy rates was likely mediated by several interacting factors that we did not assess such as interannual differences in snow depth affecting ringed seal hunting success and/or productivity (Iacozza and Ferguson, 2014), altered demographic structure such as operational and adult sex ratios (Rios Moura and Peixoto, 2013; Székely *et al.,* 2014) for the WH polar bear subpopulation, previous reproductive status, recent litter loss or cumulative effects. The WH subpopulation of polar bears is the only one where pregnancy rates are documented and offers novel insights into to a possible mechanism for climate change effects on reproduction.

Females accompanied by yearlings had elevated progesterone levels compared to females with cubs for unknown reasons. Progesterone can inhibit lactation and prevent lactose and lipid synthesis, thereby reducing milk yield and lactose concentrations (Hartmann *et al.,* 1973; Neville *et al.,* 2001). Thus, it is possible that elevated progesterone levels were related to milk production or composition (Kuhn, 1969; Derocher *et al.,* 1993a). Lactation inhibition is supported by the decrease in milk fat from 27.5% for mothers with cubs to 20.6% for females with yearlings (Derocher *et al.,* 1993a, ), resulting in lower energy transfer to yearlings (Arnould and Ramsay, 1994). Further, a lower proportion of females with yearlings lactate compared to females with cubs during the on-land period (Derocher *et al.,* 1993a). An increase in progesterone during pregnancy inhibits milk production (Wade and Schneider, 1992), and higher progesterone levels in females with yearlings may reduce lactation before weaning or be associated with cessation of lactation.

The proportion of independent yearlings declined from 81% before 1980 to 34% between 1980 and 1992 (Derocher and Stirling, 1995) declining further to 15–20% from 1991 to 1999 (Stirling *et al.,* 1999). From 1993 to 2021, we found that the proportion of yearlings independent of their mother continued to decline to 9%, which is in line with predictions from energetic models from our study area (Archer *et al.,* 2025). We found that 91% of the mothers of independent yearlings were pregnant, indicating that weaning likely occurred before or during the mating season. Weaning offspring at 1.5 years old can shorten the interbirth interval and increase reproductive output and was common during a period of population growth in WH (Ramsay and Stirling, 1988). Females that weaned yearlings ranged from 7 to 21 years old. Early weaning, however, would not increase lifetime reproductive success if cubs weaned at 16 months old have low survival. In the past, however, survival of independent yearlings and dependent yearlings was similar (Ramsay and Stirling, 1988; Derocher and Stirling, 1996). Independent yearling survival may have changed following the longer ice-free period in WH. Thus, it is unknown if the same proportion of 16-month-old offspring are being weaned and fewer are surviving, or if fewer females are weaning offspring at that age. Changes to the age at weaning may be a reproductive strategy for females throughout their life (Millar, 1977) and thought to be related to offspring size (Lee *et al.,* 1991). Timing of weaning and litter size are thought to be the only adaptable components of reproduction in mammals (Millar, 1977), and similar to a delay in primiparity possibly related to longer ice-free periods, offspring may take longer to reach optimal weaning size thus increasing the interyear birth interval.

We found that female polar bear pregnancy rates declined over time and decreased for older bears suggesting reproductive senescence. Heavier bears were more likely to be pregnant demonstrating the importance of food availability and body condition on successful reproduction. A regime shift lengthening the ice-free period in Hudson Bay may be related to a delay in first breeding consistent with life history theory. High interannual variation in pregnancy rates occurred and may be related to changing demographic structure, recent loss of offspring, or prey availability. Future research should aim to address mechanisms influencing variation in pregnancy rates.

## Data Availability

Data is available from the corresponding author upon reasonable request.

## References

[ref1] Archer L, Atkinson SN, Lunn NJ, Penk SR, Molnár PK (2025) Energetic constraints drive the decline of a sentinel polar bear population. Science 387: 516–521. 10.1126/science.adp3752.39883750

[ref2] Archer L, Atkinson SN, Pagano A, Penk S, Molnár P (2023) Lactation performance in polar bears is associated with fasting time and energetic state. Mar Ecol Prog Ser 720: 175–189. 10.3354/meps14382.

[ref3] Arnould JPY, Ramsay MA (1994) Milk production and milk consumption in polar bears during the ice-free period in western Hudson Bay. Can J Zool 72: 1365–1370. 10.1139/z94-180.

[ref4] Atkinson SN, Boulanger J, Campbell M, Trim V, Ware J, Roberto-Charron A (2022) Aerial survey of the Western Hudson Bay polar bear subpopulation 2021. Final Report. Government of Nunavut, Department of Environment, Wildlife Research Section, Status Report 2022

[ref5] Atkinson SN, Ramsay MA (1995) The effects of prolonged fasting of the body composition and reproductive success of female polar bears (*Ursus maritimus*). Funct Ecol 9: 559–567. 10.2307/2390145.

[ref6] Biddlecombe BA, Bayne EM, Lunn NJ, McGeachy D, Derocher AE (2021) Effects of sea ice fragmentation on polar bear migratory movement in Hudson Bay. Mar Ecol Prog Ser 666: 231–241. 10.3354/meps13684.

[ref8] Boyce MS (1979) Seasonality and patterns of natural selection for life histories. Am Nat 114: 569–583. 10.1086/283503.

[ref9] Burnham KP, Anderson DR (2002) Model Selection and Multimodel Inference: A Practical Information-Theoretic Approach. Springer New York, NY

[ref10] Calvert W, Ramsay MA (1998) Evaluation of age determination of polar bears by counts of cementum growth layer groups. Ursus 10: 449–453.

[ref11] Carlson DA, Gese EM (2007) Relaxin as a diagnostic tool for pregnancy in the coyote (*Canis latrans*). Anim Reprod Sci 101: 304–312. 10.1016/j.anireprosci.2006.07.011. 17069998 PMC7126177

[ref12] Castro de la Guardia L, Derocher AE, Myers PG, Terwisscha van Scheltinga AD, Lunn NJ (2013) Future sea ice conditions in Western Hudson Bay and consequences for polar bears in the 21st century. Glob Chang Biol 19: 2675–2687. 10.1111/gcb.12272. 23716301

[ref13] Cavalieri DJP, Gloersen P, Zwally HJ et al. (1996) Sea ice concentrations from Nimbus-7 SMMR and DMSP SSM/I-SSMIS passive mircowave data version 1. (1979-2019). Boulder, Colorado USA. NASA National Snow and Ice Data Center Distributed Active Archive Center

[ref14] Chambellant M, Stirling I, Gough WA, Ferguson SH (2012) Temporal variations in Hudson Bay ringed seal (*Phoca hispida*) life-history parameters in relation to environment. J Mammal 93: 267–281. 10.1644/10-MAMM-A-253.1.

[ref15] Cherry SG, Derocher AE, Thiemann GW, Lunn NJ (2013) Migration phenology and seasonal fidelity of an Arctic marine predator in relation to sea ice dynamics. J Anim Ecol 82: 912–921. 10.1111/1365-2656.12050. 23510081

[ref16] Clutton-Brock TH, Albon SD, Guinness FE (1989) Fitness costs of gestation and lactation in wild mammals. Nature 337: 260–262. 10.1038/337260a0. 2911365

[ref17] Cole LC (1954) The population consequences of life history phenomena. Q Rev Biol 29: 103–137. 10.1086/400074. 13177850

[ref19] Derocher A, Stirling I (1998) Maternal investment and factors affecting offspring size in polar bears (*Ursus maritimus*). J Zool 245: 253–260. 10.1111/j.1469-7998.1998.tb00099.x.

[ref20] Derocher AE, Andriashek D, Arnould JPY (1993a) Aspects of milk composition and lactation in polar bears. Can J Zool 71: 561–567. 10.1139/z93-077.

[ref21] Derocher AE, Andriashek D, Stirling I (1993b) Terrestrial foraging by polar bears during the ice-free period in western Hudson Bay. Arctic 46: 251–254. 10.14430/arctic1350.

[ref22] Derocher AE, Lunn NJ, Stirling I (2004) Polar bears in a warming climate. Integr Comp Biol 44: 163–176. 10.1093/icb/44.2.163. 21680496

[ref23] Derocher AE, Stirling I (1990) Distribution of polar bears (*Ursus maritimus*) during the ice-free period in western Hudson Bay. Can J Zool 68: 1395–1403. 10.1139/z90-208.

[ref24] Derocher AE, Stirling I (1992) The population dynamics of polar bears in Western Hudson Bay. In DR McCullough, RH Barrett, eds, Wildlife 2001: Populations. Springer, Netherlands, Dordrecht, pp. 1150–1159

[ref25] Derocher AE, Stirling I (1994) Age-specific reproductive performance of female polar bears (*Ursus maritimus*). J Zool 234: 527–536. 10.1111/j.1469-7998.1994.tb04863.x.

[ref26] Derocher AE, Stirling I (1995) Temporal variation in reproduction and body mass of polar bears in western Hudson Bay. Can J Zool 73: 1657–1665. 10.1139/z95-197.

[ref27] Derocher AE, Stirling I (1996) Aspects of survival in juvenile polar bears. Can J Zool 74: 1246–1252. 10.1139/z96-138.

[ref28] Derocher AE, Stirling I (1998b) Geographic variation in growth of polar bears (*Ursus maritimus*). J Zool 245: 65–72. 10.1111/j.1469-7998.1998.tb00072.x.

[ref29] Derocher AE, Stirling I, Andriashek D (1992) Pregnancy rates and serum progesterone levels of polar bears in western Hudson Bay. Can J Zool 70: 561–566. 10.1139/z92-084.

[ref30] Eberhardt LL (2002) A paradigm for population analysis of long-lived vertebrates. Ecology 83: 2841–2854. 10.1890/0012-9658(2002)083[2841:APFPAO]2.0.CO;2.

[ref31] Ferguson SH, Virgl JA, Lariviére S (1996) Evolution of delayed implantation and associated grade shifts in life history traits of North American carnivores. Ecoscience 3: 7–17.

[ref32] Ferguson SH, Young BG, Yurkowski DJ, Anderson R, Willing C, Nielsen O (2017) Demographic, ecological, and physiological responses of ringed seals to an abrupt decline in sea ice availability. PeerJ 5: e2957. 10.7717/peerj.2957. 28168119 PMC5292026

[ref33] Ferguson SH, Yurkowski DJ, Young BG, Fisk AT, Muir DCG, Zhu X, Thiemann GW (2020) Comparing temporal patterns in body condition of ringed seals living within their core geographic range with those living at the edge. Ecography 43: 1521–1535. 10.1111/ecog.04988.

[ref34] Folio DM, Aars J, Gimenez O, Derocher AE, Wiig Ø, Cubaynes S (2019) How many cubs can a mum nurse? Maternal age and size influence litter size in polar bears. Biol Lett 15: 20190070. 10.1098/rsbl.2019.0070. 31039729 PMC6548740

[ref35] Gagnon AS, Gough WA (2005) Trends in the dates of ice freeze-up and breakup over Hudson Bay, Canada. Arctic 58: 370–382.

[ref36] Gaillard J-M, Festa-Bianchet M, Yoccoz NG, Loison A, Toïgo C (2000) Temporal variation in fitness components and population dynamics of large herbivores. Annu Rev Ecol Evol Syst 31: 367–393. 10.1146/annurev.ecolsys.31.1.367.

[ref37] de Haas van Dorsser FJ, Swanson WF, Lasano S, Steinetz BG (2006) Development, validation, and application of a urinary relaxin radioimmunoassay for the diagnosis and monitoring of pregnancy in felids. Biol Reprod 74: 1090–1095. 10.1095/biolreprod.105.050146. 16481588

[ref38] Hartmann P, Trevethan P, Shelton J (1973) Progesterone and oestrogen and the initiation of lactation in ewes. J Endocrinol 59: 249–259. 10.1677/joe.0.0590249. 4759591

[ref39] Hobson KA, Stirling I, Andriashek DS (2009) Isotopic homogeneity of breath CO_2_ from fasting and berry-eating polar bears: implications for tracing reliance on terrestrial foods in a changing Arctic. Can J Zool 87: 50–55. 10.1139/Z08-137.

[ref40] Hochheim K, Barber D, Lukovich J (2010) Changing sea ice conditions in Hudson Bay, 1980–2005. In SH Ferguson, LL Loseto, ML Mallory, eds, A Little Less Arctic: Top Predators in the World's Largest Northern Inland Sea, Hudson Bay. Springer, London, New York, pp. 39–52

[ref41] Iacozza J, Ferguson SH (2014) Spatio-temporal variability of snow over sea ice in western Hudson Bay, with reference to ringed seal pup survival. Polar Biol 37: 817–832. 10.1007/s00300-014-1484-z.

[ref42] Johnson AC, Hobson KA, Lunn NJ, McGeachy D, Richardson ES, Derocher AE (2019) Temporal and intra-population patterns in polar bear foraging ecology in western Hudson Bay. Mar Ecol Prog Ser 619: 187–199. 10.3354/meps12933.

[ref43] Johnson AC, Reimer JR, Lunn NJ, Stirling I, McGeachy D, Derocher AE (2020) Influence of sea ice dynamics on population energetics of Western Hudson Bay polar bears. Consn Physiol 8: coaa132. 10.1093/conphys/coaa132. PMC7772618 33408870

[ref44] Jonkel CJ, Kolenosky GB, Robertson RJ, Russell RH (1972) Further notes on polar bear denning habits. Bears: Their Biol Manage 2: 142–158. 10.2307/3872578.

[ref45] Kendall MG (1975) Rank correlation methods. Griffin, London. J Econom 13: 245–259.

[ref46] Kuhn NJ (1969) Progesterone withdrawal as the lactogenic trigger in the rat. J Endocrinol 44: 39–54. 10.1677/joe.0.0440039. 5814248

[ref47] Lake S, Burton H, Barker R, Hindell M (2008) Annual reproductive rates of Weddell seals in eastern Antarctica from 1973 to 2000. Mar Ecol Prog Ser 366: 259–270. 10.3354/meps07502.

[ref48] Lee PC, Majluf P, Gordon IJ (1991) Growth, weaning and maternal investment from a comparative perspective. J Zool 225: 99–114. 10.1111/j.1469-7998.1991.tb03804.x.

[ref49] Lunn NJ, Boyd IL, Croxall JP (1994) Reproductive performance of female Antarctic fur seals: the influence of age, breeding experience, environmental variation and individual quality. J Anim Ecol 63: 827–840. 10.2307/5260.

[ref50] Lunn NJ, Servanty S, Regehr EV, Converse SJ, Richardson E, Stirling I (2016) Demography of an apex predator at the edge of its range: impacts of changing sea ice on polar bears in Hudson Bay. Ecol Appl 26: 1302–1320. 10.1890/15-1256. 27755745

[ref51] Lunn NJ, Stirling I, Andriashek D, Richardson E (2004) Selection of maternity dens by female polar bears in western Hudson Bay, Canada and the effects of human disturbance. Polar Biol 27: 350–356. 10.1007/s00300-004-0604-6.

[ref52] Malenfant RM, Coltman DW, Richardson ES, Lunn NJ, Stirling I, Adamowicz E, Davis CS (2016) Evidence of adoption, monozygotic twinning, and low inbreeding rates in a large genetic pedigree of polar bears. Polar Biol 39: 1455–1465. 10.1007/s00300-015-1871-0.

[ref53] Mann HB (1945) Nonparametric tests against trend. Econometrica: J 13: 245. 10.2307/1907187.

[ref54] McCall AG, Derocher AE, Lunn NJ (2015) Home range distribution of polar bears in western Hudson Bay. Polar Biol 38: 343–355. 10.1007/s00300-014-1590-y.

[ref55] McGeachy D, Lunn NJ, Derocher AE (2024a) Possible sexually selected infanticide by polar bears in western Hudson Bay. Ursus 2024: 11. 10.2192/URSUS-D-23-00028.

[ref56] McGeachy D, Lunn NJ, Richardson ES, Derocher AE (2024b) Sea ice influence on male polar bear survival in Hudson Bay. Arctic Sci 10: 483–498. 10.1139/as-2023-0045.

[ref57] Medill S, Derocher AE, Stirling I, Lunn N (2010) Reconstructing the reproductive history of female polar bears using cementum patterns of premolar teeth. Polar Biol 33: 115–124. 10.1007/s00300-009-0689-z.

[ref58] Millar JS (1977) Adaptive features of mammalian reproduction. Evolution 31: 370–386. 10.2307/2407759. 28563222

[ref59] Miller EN, Lunn NJ, McGeachy D, Derocher AE (2022) Autumn migration phenology of polar bears (*Ursus maritimus*) in Hudson Bay, Canada. Polar Biol 45: 1023–1034. 10.1007/s00300-022-03050-3.

[ref60] Molnár PK, Bitz CM, Holland MM, Kay JE, Penk SR, Amstrup SC (2020) Fasting season length sets temporal limits for global polar bear persistence. Nat Clim Chang 10: 732–738. 10.1038/s41558-020-0818-9.

[ref61] Murphy B (2012) Embryonic diapause: advances in understanding the enigma of seasonal delayed implantation. Repro Domest Anim 47: 121–124. 10.1111/rda.12046. 23279480

[ref62] Naciri M, Aars J, Blanchet M-A, Gimenez O, Cubaynes S (2022) Reproductive senescence in polar bears in a variable environment. Front Ecol and Evol 10: 920481. 10.3389/fevo.2022.920481.

[ref63] Neville MC, Morton J, Umemura S (2001) Lactogenesis: the transition from pregnancy to lactation. Ped Clin North Am 48: 35–52. 10.1016/S0031-3955(05)70284-4. 11236732

[ref64] Northrup JM, Howe E, Lunn NJ, Middel K, Obbard ME, Ross TR, Guillaume S, Walton L, Ware J (2022) 2021 Southern Hudson Bay polar bear subpopulation aerial survey. Ontario Ministry of Natural Resouces, Wildlife Research and Montiroting Section, Final report, p. 52

[ref65] Pagano AM, Rode KD, Lunn NJ, McGeachy D, Atkinson SN, Farley SD, Erlenbach JA, Robbins CT (2024) Polar bear energetic and behavioral strategies on land with implications for surviving the ice-free period. Nat Commun 15: 947. 10.1038/s41467-023-44682-1. 38351211 PMC10864307

[ref66] Pilfold NW, Hedman D, Stirling I, Derocher AE, Lunn NJ, Richardson E (2016) Mass loss rates of fasting polar bears. Physiol Biochem Zool 89: 377–388. 10.1086/687988. 27617359

[ref67] R Development Core Team (2021) R; A Language and Environment for Statistical Computing R Foundation for Statistical Computing, Vienna, Austria

[ref68] Ramsay MA, Andriashek DS (1986) Long distance route orientation of female polar bears (*Ursus maritimus*) in spring. J Zool 208: 63–72. 10.1111/j.1469-7998.1986.tb04709.x.

[ref69] Ramsay MA, Stirling I (1984) Interactions of wolves and polar bears in northern Manitoba. J Mammal 65: 693–694. 10.2307/1380856.

[ref70] Ramsay MA, Stirling I (1986) On the mating system of polar bears. Can J Zool 64: 2142–2151. 10.1139/z86-329.

[ref71] Ramsay MA, Stirling I (1988) Reproductive biology and ecology of female polar bears (*Ursus maritimus*). J Zool 214: 601–633. 10.1111/j.1469-7998.1988.tb03762.x.

[ref72] Regehr EV, Lunn NJ, Amstrup SC, Stirling IAN (2007) Effects of earlier sea ice breakup on survival and population size of polar bears in western Hudson Bay. J Wildl Manage 71: 2673–2683. 10.2193/2006-180.

[ref73] Renfree MB, Fenelon JC (2017) The enigma of embryonic diapause. Development 144: 3199–3210. 10.1242/dev.148213. 28928280

[ref74] Rios Moura R, Peixoto PEC (2013) The effect of operational sex ratio on the opportunity for sexual selection: a meta-analysis. Anim Behav 86: 675–683. 10.1016/j.anbehav.2013.07.002.

[ref75] Robbins CT, Ben-David M, Fortin JK, Nelson OL (2012a) Maternal condition determines birth date and growth of newborn bear cubs. J Mammal 93: 540–546. 10.1644/11-MAMM-A-155.1.

[ref76] Robbins CT, Lopez-Alfaro C, Rode KD, Tøien Ø, Nelson OL (2012b) Hibernation and seasonal fasting in bears: the energetic costs and consequences for polar bears. J Mammal 93: 1493–1503. 10.1644/11-MAMM-A-406.1.

[ref77] Roff DA (1992) The Evolution of Life Histories: Theory and Analysis. Chapman & Hall, New York

[ref78] Russell RH (1975) The food habits of polar bears of James Bay and Southwest Hudson Bay in summer and autumn. Arctic 28: 117–129. 10.14430/arctic2823.

[ref79] Sandell M (1990) The evolution of seasonal delayed implantation. Q Rev Biol 65: 23–42. 10.1086/416583. 2186428

[ref80] Sciullo L, Thiemann GW, Lunn NJ (2016) Comparative assessment of metrics for monitoring the body condition of polar bears in western Hudson Bay. J Zool 300: 45–58. 10.1111/jzo.12354.

[ref81] Scott J, Marshall G (2010) A step-change in the date of sea-ice breakup in western Hudson Bay. Arctic 63: 155–164. 10.14430/arctic971.

[ref82] Shimozuru M, Iibuchi R, Yoshimoto T, Nagashima A, Tanaka J, Tsubota T (2013) Pregnancy during hibernation in Japanese black bears: effects on body temperature and blood biochemical profiles. J Mammal 94: 618–627. 10.1644/12-MAMM-A-246.1.

[ref83] Stearns SC (1989) Trade-offs in life-history evolution. Funct Ecol 3: 259–268. 10.2307/2389364.

[ref84] Stern HL, Laidre KL (2016) Sea-ice indicators of polar bear habitat. Cryosphere 10: 2027–2041. 10.5194/tc-10-2027-2016.

[ref85] Stirling I (2005) Reproductive rates of ringed seals and survival of pups in northwestern Hudson Bay, Canada, 1991–2000. Polar Biol 28: 381–387. 10.1007/s00300-004-0700-7.

[ref86] Stirling I, Archibald WR (1977) Aspects of predation of seals by polar bears. J Fish Res Board Can 34: 1126–1129. 10.1139/f77-169.

[ref87] Stirling I, Derocher AE (1993) Possible impacts of climatic warming on polar bears. Arctic 46: 240–245. 10.14430/arctic1348.

[ref88] Stirling I, Jonkel C, Robertson R et al. (1977) The ecology of the polar bear (*Ursus maritimus*) along the western coast of Hudson Bay. Can Wildl Serv Occas Pap 33: 1–64.

[ref89] Stirling I, Lunn N, Iacozza J, Elliott C, Obbard M (2004) Polar bear distribution and abundance on the southwestern Hudson Bay coast during open water season, in relation to population trends and annual ice patterns. Arctic 57: 15–26. 10.14430/arctic479.

[ref90] Stirling I, Lunn NJ, Iacozza J (1999) Long-term trends in the population ecology of polar bears in western Hudson Bay in relation to climatic change. Arctic 52: 294–306. 10.14430/arctic935.

[ref91] Stirling I, Spencer C, Andriashek D (1989) Immobilization of polar bears (*Ursus maritimus*) with Telazol in the Canadian Arctic. J Wildl Dis 25: 159–168. 10.7589/0090-3558-25.2.159. 2716095

[ref104] Stirling I, Thiemann GW, Richardson, ES (2008). Quantitative support for a subjective fatness index for immobilized polar bears. J Wildl Manage 72: 568–574.

[ref92] Støen O-G, Zedrosser A, Wegge P, Swenson JE (2006) Socially induced delayed primiparity in brown bears Ursus arctos. Behav Ecol Sociobiol 61: 1–8. 10.1007/s00265-006-0231-z.

[ref93] Stroeve J, Crawford C, Ferguson S, Stirling S, Archer L, York G, Babb D, Mallett R (2024) Ice-free period too long for southern and Western Hudson Bay polar bear populations if global warming exceeds 1.6 to 2.6 °C. Commun Earth Environ 5: 296. 10.1038/s43247-024-01430-7.

[ref94] Székely T, Weissing FJ, Komdeur J (2014) Adult sex ratio variation: implications for breeding system evolution. J Evol Biol 27: 1500–1512. 10.1111/jeb.12415. 24848871

[ref95] Tavecchia G, Coulson T, Morgan BJT, Pemberton JM, Pilkington JC, Gulland FMD, Clutton-Brock TH (2005) Predictors of reproductive cost in female Soay sheep. J Anim Ecol 74: 201–213. 10.1111/j.1365-2656.2005.00916.x.

[ref96] Taylor MK, Laake J, McLoughlin PD, Cluff HD, Messier F (2008) Mark-recapture and stochastic population models for polar bears of the high Arctic. Arctic 61: 143–152.

[ref97] Thiemann GW, Iverson SJ, Stirling I (2008) Polar bear diets and arctic marine food webs: insights from fatty acid analysis. Ecological monographs 78: 591–613. 10.1890/07-1050.1.

[ref98] Thiemann GW, Lunn NJ, Richardson ES, Andriashe KDS (2011) Temporal change in the morphometry—body mass relationship of polar bears. J Wildl Manage 75: 580–587. 10.1002/jwmg.112.

[ref99] Thom MD, Johnson DDP, Macdonald DW (2004) The evolution and maintenance of delayed implantation in the mustelidae (Mammalia: Carnivora). Evol 58: 175–183. 10.1111/j.0014-3820.2004.tb01584.x 15058730

[ref100] Tiwari RC, Cronin KA, Davis W, Feuer EJ, Yu B, Chib S (2005) Bayesian model selection for join point regression with application to age-adjusted cancer rates. J R Stat Soc Ser C Appl Stat 54: 919–939. 10.1111/j.1467-9876.2005.00518.x.

[ref101] Wade GN, Schneider JE (1992) Metabolic fuels and reproduction in female mammals. Neurosci Biobehav Rev 16: 235–272. 10.1016/S0149-7634(05)80183-6. 1630733

[ref102] Wightman NE, Howe E, Satura A, Northrup JM (2022) Factors affecting age at primiparity in black bears. J Wildl Manage 86: e22297. 10.1002/jwmg.22297.

[ref103] Young BG, Ferguson SH, Lunn NJ (2015) Variation in ringed seal density and abundance in Western Hudson Bay estimated from aerial surveys, 1995 to 2013. Arctic 68: 301–309. 10.14430/arctic4503.

